# Comparison of acute kidney injury between open and laparoscopic pylorus-preserving pancreaticoduodenectomy: Propensity score analysis

**DOI:** 10.1371/journal.pone.0202980

**Published:** 2018-08-24

**Authors:** Yong-Seok Park, In-Gu Jun, Yonji Go, Jun-Gol Song, Gyu-Sam Hwang

**Affiliations:** Department of Anesthesiology and Pain Medicine, Laboratory for Cardiovascular Dynamics, Asan Medical Center, University of Ulsan College of Medicine, Seoul, Korea; University of Sao Paulo Medical School, BRAZIL

## Abstract

Laparoscopic pylorus-preserving pancreaticoduodenectomy is being performed more frequently because of improved surgical techniques. Although several studies have demonstrated safety and favourable outcomes of laparoscopic pylorus-preserving pancreaticoduodenectomy compared to open pylorus-preserving pancreaticoduodenectomy, few studies have focused on the development of postoperative acute kidney injury. This retrospective study compared the prevalence and risk factors of acute kidney injury following laparoscopic and open pylorus-preserving pancreaticoduodenectomy. Data from 809 patients who underwent pylorus-preserving pancreaticoduodenectomy between February 2012 and September 2016 were analysed. Patients were divided into two groups according to the surgical procedure (open pylorus-preserving pancreaticoduodenectomy [n = 632] vs laparoscopic pylorus-preserving pancreaticoduodenectomy [n = 177]). The Kidney Disease: Improving Global Outcomes criteria were used to define postoperative acute kidney injury and risk factors were investigated using multivariable logistic regression analysis with propensity score matching analysis and standardized mortality ratio weighting to compare outcomes. No significant differences were found in the occurrence of postoperative acute kidney injury and incidence of postoperative ICU admission between open and laparoscopic pylorus-preserving pancreaticoduodenectomy groups after propensity score matching (p = 1.000, p = 0.999, respectivelyand standardized mortality ratio weighted analysis (p = 0.619, p = 0.982, respectively). Hospital stay was significantly shorter in the laparoscopic pylorus-preserving pancreaticoduodenectomy group (propensity matched set, mean [SD], 16.7 [10.0] vs. 18.7 [9.6] days, p = 0.004; standardized mortality ratio, 16.6 [9.9] vs. 18.1 [8.8] days, p = 0.001). There was no significant difference in postoperative acute kidney injury incidence between both groups. Laparoscopic pylorus-preserving pancreaticoduodenectomy is promising with comparable postoperative outcomes to open pylorus-preserving pancreaticoduodenectomy and has the advantage of shorter hospital stay.

## Introduction

With the improvement of surgical techniques, the scope of minimally invasive surgery has also expanded. Laparoscopic pylorus-preserving pancreaticoduodenectomy (LPPPD) has been reported to be safe and feasible with acceptable outcomes despite its technical difficulties [1, 2]. Studies comparing LPPPD with open pylorus-preserving pancreaticoduodenectomy (OPPPD) have addressed either favorable oncological outcomes or surgical outcomes to date [3, 4].

Acute kidney injury (AKI) is known to be associated with poor postoperative outcomes including short-term and long-term mortality [5–7]. AKI is a common complication following cardiac surgery and is associated with increased long-term mortality in coronary artery bypass graft surgery and cardiac valve operations [7–10]. In case of noncardiac surgery, the incidence of AKI is approximately 1%, which increases up to 32% after major surgery, and is also significantly associated with high mortality [6, 11, 12]. Furthermore, AKI episodes with a slight increase in serum creatinine (sCr) have been reported to increase long-term mortality after major surgery, even if renal function was restored during the hospitalisation [6].

In laparoscopic surgery, relatively less surgical trauma and systemic inflammatory responses may influence the incidence of AKI [13, 14]. However, few studies have focused on the development of postoperative AKI in patients undergoing LPPPD or OPPPD, whereas some researchers have reported a lower incidence of AKI after robot-assisted laparoscopic radical prostatectomy and laparoscopic liver resection than after open surgery [14, 15]. Therefore, the aim of the present study was to compare the incidence of postoperative AKI in patients undergoing LPPPD or OPPPD. In addition, we investigated postoperative outcomes such as incidence of ICU admission and hospital stay after LPPPD and OPPPD.

## Methods

**Fig 1:**
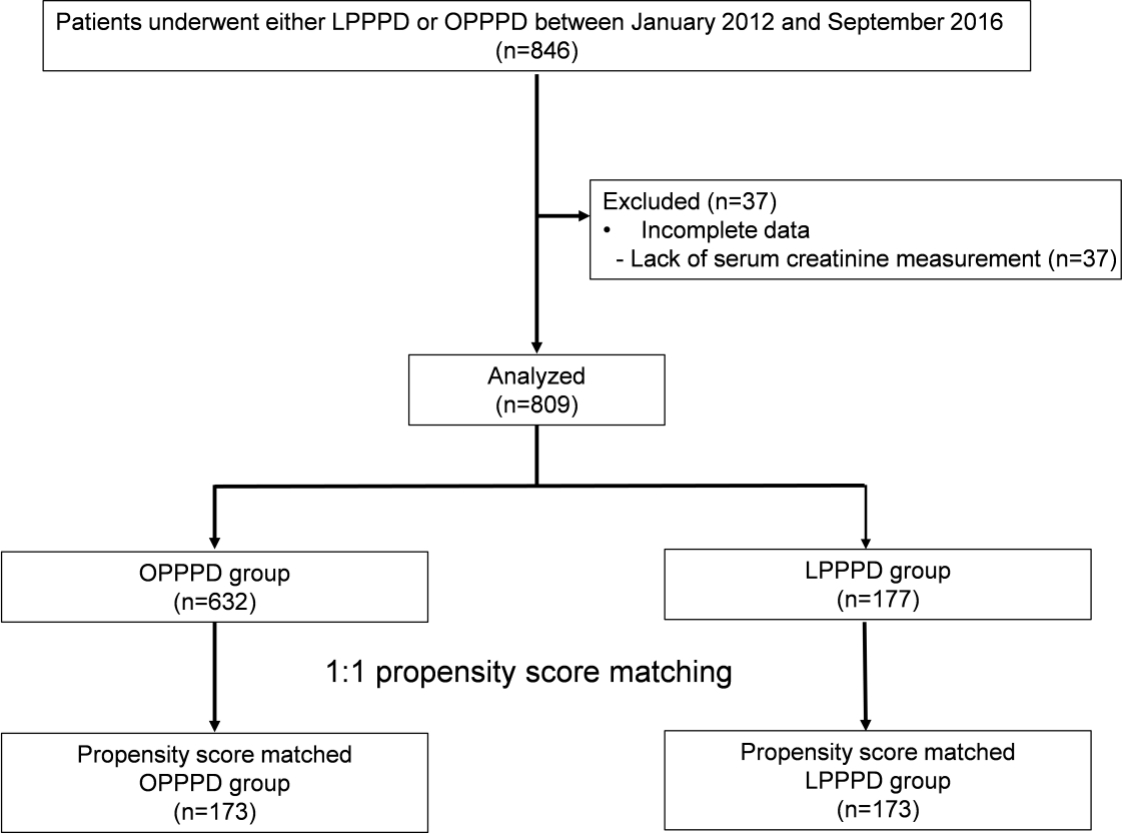
Study flow diagram. OPPPD, open pylorus-preserving pancreaticoduodenectomy; LPPPD, laparoscopic pylorus-preserving pancreaticoduodenectomy.

This study was approved by our institutional review board. (Asan Medical Center Institutional Review Board). The informed consent was waived by the institutional review board because of the minimal risk of this retrospective study. We retrospectively collected the data of patients who underwent LPPPD or OPPPD at our centre from February 2012 to September 2016. Patients with incomplete data or including missing sCr values were excluded (Fig 1).

### Data collection

All data including baseline characteristics, laboratory values, intraoperative information, and outcome data of study patients were collected using a computerized patient data recording system (Asan Biomedical Research Program, ABLE, South Korea). Sex, age, height, weight, body mass index (BMI), underlying disease such as diabetes mellitus, hypertension, and cerebrovascular disease, and current medications including angiotensin-converting enzyme inhibitors, calcium channel blockers, beta blockers, and statin, were included in the baseline characteristics. Laboratory data included determination of levels of hemoglobin, platelets, aspartate transaminase, alanine transaminase, total bilirubin, total protein, albumin, serum creatinine, glucose, and sodium in addition to prothrombin time. Intraoperative data included administered fluid volumes such as crystalloid, synthetic colloid, albumin, and transfused blood products such as packed red blood cell (RBC) and fresh-frozen plasma, total urine output, lowest mean arterial pressure (MAP) recorded during the operation, and infusion of inotropes or vasopressors.

### Anesthetic technique

Anesthesia was performed according to our routine protocols. No premedication was given to the patients. Electrocardiography, pulse oximetry, capnography, and noninvasive blood pressure were monitored from the induction of anesthesia to the end of surgery. General anesthesia was induced with propofol (2 mg.kg^-1^) and rocuronium (0.6–1.2 mg.kg^-1^) and maintained with sevoflurane (1–3 vol%) and target-controlled infusion of remifentanil (2–5 ng.ml^-1^). After induction, arterial catheterisation was performed for continuous blood pressure monitoring and the central venous catheter was inserted into the internal jugular vein to infuse fluid and monitor central venous pressure. Crystalloids including Ringer’s lactate or PlasmaLyte were infused as maintenance fluid and synthetic colloids such as Voluven (Voluven^®^; Fresenius Kabi, Bad Homburg, Germany) or 5% albumin were administered for volume replacement at the discretion of the anesthesiologist. Appropriate packed RBC was transfused to maintain target hemoglobin level ≥ 80 g.l^-1^ in patients without history of ischemic heart disease and cerebrovascular disease, and at ≥ 100 g.l^-1^ in patients with history of ischemic heart disease or cerebrovascular disease. Inotropes or vasopressors were administered if the MAP was <65 mmHg.

### Surgical technique

Surgical procedures followed the routine protocols established at our centre [4]. A patient undergoing LPPPD was placed in supine position and the reverse Trendelenburg position. A 12-mm trocar was placed to establish the pneumoperitoneum, and three or four more trocars were placed. Intraabdominal CO_2_ gas pressure was maintained at about 12 mmHg. In a patient undergoing OPPPD, an approach to the surgical field was established by an inverted L or long midline incision, and the same process of reconstruction as LPPPD was performed [2, 4].

### Outcomes

The development of postoperative AKI was the primary outcome of this study. The postoperative AKI was defined by the Kidney Disease: Improving Global Outcomes (KDIGO) criteria; an increase of ≥ 0.3 mg.dl^-1^ in sCr within postoperative 2 days, an increase in sCr ≥ 1.5 times from baseline within postoperative seven days [16]. Other outcomes including hospital stay and postoperative ICU stay were evaluated.

### Statistical analysis

Continuous variables were expressed as the mean [SD] or median [IQR]. Continuous variables were compared using Student *t*-test or Mann-Whitney U test. Categorical variables are reported as frequencies and proportions and were analysed using the Chi-square test or the Fisher’s exact test, as appropriate. Multiple logistic regression analysis was used to detect factors associated with AKI and all variables with p<0.1 in univariate analysis were included in the multivariable analysis. Stepwise regression analysis was done to select variables accepted in the multivariate model.

The propensity score (PS) was estimated with groups as the dependent variables by multiple logistic regression analysis. A full non-parsimonious model including demographics and preoperative variables in Table 1 was developed. Intraoperative variables were not included in the model. Model discrimination was assessed with C-statistic (= 0.769), and model calibration was assessed with the Hosmer-Lemeshow statistic (χ^2^ = 8.6581, DF = 8, p = 0.372). PS matching was performed by Greedy matching using a caliper of 0.25 SDs of the logit of the PS. After PS matching, baseline variables were compared between the two groups. Categorical variables were compared using the McNemar test, and continuous variables were compared using the paired t-test or Wilcoxon signed-rank test as appropriate. In addition, we performed a weighted PS analysis using standardized mortality ratio (SMR) weighting. In terms of the average treatment effect on the treated (ATT), the weights for the OPPPD group were PS/(1—PS), and weights for patients receiving LPPPD were 1. The absolute standardized differences were used to diagnose the balance after propensity analysis. All absolute standardized differences after matching were less than 0.1. In the PS matched cohort, the risks of each outcome were compared with logistic regression using Generalized Estimating Equations that accounted for the clustering of matched pairs. Additionally, the outcomes were evaluated using weighted regression models with PS matching and SMR weighting.

**Table 1:** Patient demographics and perioperative variables. Values are expressed as the mean (SD), median (interquartile range), or n (proportion).

		Total (n = 809)	OPPPD (n = 632)	LPPPD (n = 177)	p value
**Demographics**					
	Age; y	60.2 (11.9)	61.3 (11.0)	56.1 (13.8)	<0.001
	Sex; male	458 (56.6)	382 (60.4)	76 (42.9)	<0.001
	BMI; kg.m^-2^	23.6 (3.3)	23.9 (3.5)	22.4 (2.4)	<0.001
	Diabetes	181 (22.4)	160 (25.3)	21 (11.9)	<0.001
	Hypertension	296 (36.6)	255 (40.4)	41 (23.2)	<0.001
	Cerebrovascular disease	54 (6.7)	47 (7.4)	7 (4.0)	0.101
**Medications**					
	ACE inhibitor	116 (14.3)	94 (14.9)	22 (12.4)	0.412
	Calcium channel blocker	137 (16.9)	116 (18.4)	21 (11.9)	0.042
	Beta blocker	54 (6.7)	48 (7.6)	6 (3.4)	0.048
	statin	76 (9.4)	62 (9.8)	14 (7.9)	0.444
**Preoperative variables**					
	Hemoglobin; g.dl^-1^	126 (16)	126 (16)	127 (15)	0.974
	Platelets; ×10^3^. μl^-1^	246.7 (76.9)	246.1 (79.1)	248.7 (68.9)	0.194
	Prothrombin time; INR	0.99 (0.08)	1.00 (0.08)	0.99 (0.07)	0.521
	Creatinine; mg.dl^-1^	0.8 (0.3)	0.8 (0.4)	0.7 (0.2)	0.049
	Albumin; g.dl^-1^	3.6 (0.5)	3.5 (0.5)	3.7 (0.4)	<0.001
	Total bilirubin; mg.dl^-1^	1.4 (2.3)	1.6 (2.5)	0.9 (1.5)	<0.001
	AST; IU.l^-1^	32.3 (29.3)	33.8 (31.1)	26.9 (20.5)	<0.001
	ALT; IU.l^-1^	45.0 (60.1)	48.9 (62.8)	30.9 (46.5)	<0.001
	Sodium; mmol.l^-1^	139.9 (2.9)	139.8 (3.0)	140.3 (2.6)	0.054
	Glucose; mg.dl^-1^	135.1 (62.7)	137.7 (62.7)	125.6 (62.2)	<0.001
**Intraoperative variables**					
	Crystalloid; ml	3403 (1202)	3359 (1227)	3559 (1096)	0.051
	Synthetic colloid; ml	664 (412)	702 (391)	529 (457)	<0.001
	Albumin infusion; ml	160 (296)	151 (300)	195 (281)	0.076
	RBC transfusion; units	0.4 (1.0)	0.4 (1.1)	0.2 (0.7)	0.011
	Lowest MAP	59 (6.2)	59 (6.2)	61 (6.1)	0.001
	Urine output; ml	503 (334)	499 (347)	519 (282)	0.423
	Duration of surgery; min	436 (87)	423 (83)	484 (83)	<0.001
**Diagnosis**					**<**0.001
	Pancreatic ductal adenocarcinoma	281 (34.7)	266 (42.1)	15 (8.5)	
	IPMN	154 (19.0)	104 (16.5)	50 (28.3)	
	Distal common bile duct cancer	121 (15.0)	103 (16.3)	18 (10.2)	
	Ampulla of Vater cancer	105 (13.0)	74 (11.7)	31 (17.5)	
	PNET	41 (5.1)	21 (3.3)	20 (11.3)	
	SPN	36 (4.5)	13 (2.1)	23 (13.0)	
	Duodenal cancer	15 (1.9)	12 (1.9)	3 (1.7)	
	Others	15 (1.9)	12 (1.9)	3 (1.7)	

OPPPD, open pylorus-preserving pancreaticoduodenectomy; LPPPD, laparoscopic pylorus-preserving pancreaticoduodenectomy; BMI, body mass index; ACE, angiotensin converting enzyme, AST, aspartate transaminase; ALT, alanine transaminase; MAP, mean arterial pressure; IPMN, intraductal papillary mucinous neoplasm; PNET, pancreatic neuroendocrine tumors; SPN, solid pseudopapillary neoplasm.

## Results

Of the 846 patients enrolled, 37 patients were excluded due to incomplete data; thus, a total of 809 patients were included in this study (Fig 1). Of these, 632 patients underwent OPPPD and 177 patients underwent LPPPD. Baseline characteristics and perioperative variables of these patients are shown in Table 1. The most common diagnosis for surgery was pancreatic ductal adenocarcinoma (281/809, 34.7%), followed by intraductal papillary mucinous neoplasm (154/809, 19.0%), distal common bile duct cancer (121/809, 15.0%), and ampulla of Vater cancer (105/809, 13.0%). The patients who underwent LPPPD were younger (p<0.001), women (p<0.001), had a lower BMI (p<0.001), and had a lower incidence of diabetes mellitus (p<0.001) and hypertension (p<0.001). During the operation, the LPPPD group received lower amounts of synthetic colloid (p<0.001) and fewer units of packed RBC (p = 0.011). Operative time of LPPPD was longer than OPPPD (p<0.001). The overall AKI incidence was 5.3% (43/809).

After 1:1 PS analysis, all absolute standardized differences in demographics or preoperative laboratory variables after matching were less than 0.1 between the OPPPD (n = 173) and LPPPD groups (n = 173) (Table 2). After PS-matched analysis, synthetic colloids were more frequently administered to patients undergoing OPPPD (148/173, 85.6%) than LPPPD (110/173, 63.6%) (p<0.001), while the incidence of albumin infusion was higher in the LPPPD group than in the OPPPD group (52.6% vs. 39.3%; p = 0.013).

**Table 2:** Patient demographics and baseline variables after propensity score matching. Values are expressed as the mean (SD), median (interquartile range), or n (proportion).

		OPPPD (n = 173)	LPPPD (n = 173)	p value	Standardized difference
**Demographics**					
	Age; y	56.5 (12.0)	56.5 (13.7)	0.985	0.002
	Sex; male	81 (46.8)	76 (43.9)	0.569	0.058
	BMI; kg.m-^2^	22.5 (3.0)	22.5 (2.4)	0.895	0.013
	Diabetes	21 (12.1)	21 (12.1)	1.000	0
	Hypertension	45 (26.0)	41 (23.7)	0.606	0.055
	Cerebrovascular disease	9 (5.2)	7 (4.1)	0.617	0.059
**Medications**					
	ACE inhibitor	26 (15.0)	22 (12.7)	0.537	0.070
	Calcium channel blocker	23 (13.3)	21 (12.1)	0.739	0.036
	Beta blocker	9 (5.2)	6 (3.5)	0.405	0.096
	Statin	16 (9.3)	14 (8.1)	0.715	0.043
**Preoperative variables**					
	Hemoglobin; g.dl^-1^	126 (15)	126 (16)	0.899	0.012
	Platelets; ×10^3^.μl^-1^	250.9 (72.8)	248.9 (69.2)	0.799	0.028
	Prothrombin time; INR	0.99 (0.07)	0.99 (0.07)	0.716	0.034
	Creatinine; mg.dl^-1^	0.7 (0.2)	0.7 (0.2)	0.544	0.062
	Albumin; g.dl^-1^	3.7 (0.4)	3.7 (0.4)	0.957	0.006
	Total bilirubin; mg.dl^-1^	1.0 (1.2)	0.9 (1.5)	0.101	0.007
	AST; IU.l^-1^	27.3 (20.7)	27.1 (20.6)	0.614	0.009
	ALT; IU.l^-1^	31.3 (37.2)	31.1 (47.0)	0.261	0.005
	Sodium; mmol.l^-1^	140.1 (3.3)	140.2 (2.6)	0.744	0.039
	Glucose; mg.dl^-1^	127.2 (57.1)	126.1 (62.8)	0.604	0.018

OPPPD, open pylorus-preserving pancreaticoduodenectomy; LPPPD, laparoscopic pylorus-preserving pancreaticoduodenectomy; BMI, body mass index; ACE, angiotensin converting enzyme, AST, aspartate transaminase; ALT, alanine transaminase.

In the multivariable analysis, LPPPD was not significantly associated with the development of postoperative AKI (odds ratio [OR] 0.932, 95% confidence interval [95%CI] 0.371–2.343, p = 0.880). Conversely, hypertension (OR 2.400, 95%CI 1.180–4.883, p = 0.016), preoperative albumin level (OR 0.488, 95%CI 0.242–0.987, p = 0.046), and synthetic colloids infusion (OR 4.871, 95%CI 1.122–21.148, p = 0.035) were significantly associated with AKI after surgery (Table 3).

**Table 3:** Multivariable analysis of risk factors associated with postoperative acute kidney injury.

	Univariate			Multivariable			
	Odds ratio	95%CI	p value	Odds ratio	95% CI		p value
**LPPPD**	0.564	0.234–1.359	0.202	0.932	0.371–2.343		0.880
**Sex**	1.822	0.936–3.548	0.077				
**Age**	1.041	1.010–1.072	0.010	1.011	0.976–1.047		0.545
**BMI**	1.060	0.973–1.155	0.183				
**Diabetes**	1.732	0.895–3.352	0.103	1.039	0.512–2.107		0.916
**Hypertension**	3.469	1.821–6.608	0.000	2.400	1.180–4.883		0.016
**Hemoglobin**	0.743	0.612–0.902	0.003				
**Platelets**	1.001	0.998–1.005	0.486				
**Glucose**	0.997	0.986–1.008	0.568				
**Albumin**	0.359	0.187–0.690	0.002	0.488	0.242–0.987		0.046
**AST**	1.002	0.993–1.012	0.648				
**ALT**	0.998	0.992–1.004	0.552				
**Total bilirubin**	1.133	1.044–1.230	0.003				
**Crystalloid**	1.000	1.000–1.000	0.670				
**Synthetic colloid**	5.541	1.326–23.152	0.019	4.871	1.122–21.148		0.035
**Transfusion**	1.929	0.965–3.858	0.063				
**Urine output**^a^	0.999	0.998–1.000	0.113				
**Lowest MAP**	0.934	0.886–0.985	0.012	0.962	0.909–1.019		0.186
**Vasopressor use**	1.566	0.844–2.907	0.155				

LPPPD, Laparoscopic pylorus-preserving pancreaticoduodenectomy; BMI, body mass index; AST, aspartate transaminase; ALT, alanine transaminase; SBP, systolic blood pressure; DBP, diastolic blood pressure.

^a^Total intraoperative urine output.

**Fig 2:**
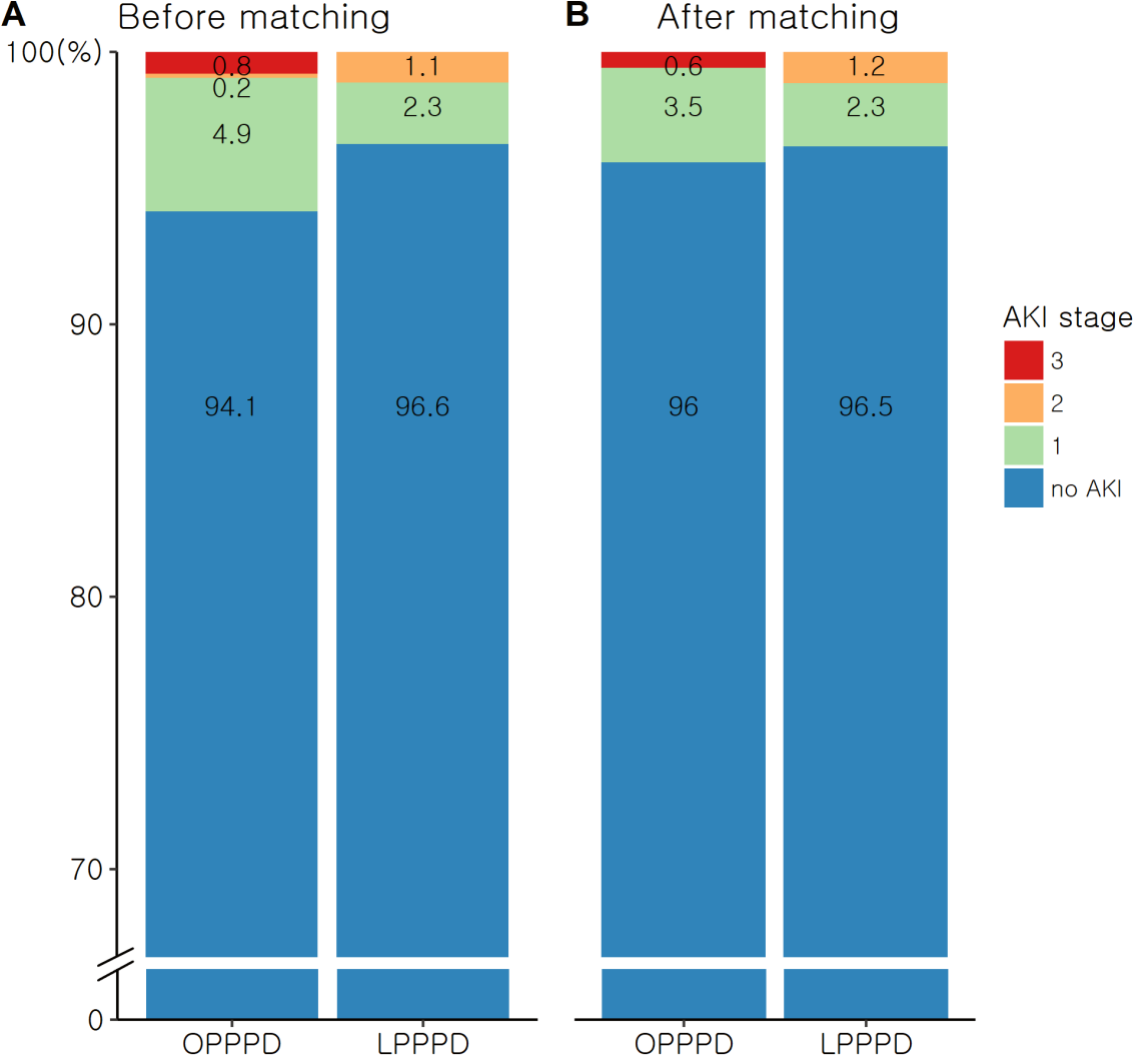
Proportions of acute kidney injury stages by Kidney Disease: Improving Global Outcomes (KDIGO) criteria in the open pylorus-preserving pancreaticoduodenectomy (OPPPD) group and the laparoscopic pylorus-preserving pancreaticoduodenectomy (LPPPD) group. (A) Before and (B) after propensity score matching analysis. AKI, acute kidney injury.

Comparing the outcomes of the OPPPD and LPPPD group, there were no significant differences in the occurrence of postoperative AKI (OPPPD group, 37/632 [5.9%], KDIGO stage 1/2/3, 31/1/5; LPPPD group, 6/177 [3.4%], KDIGO stage 1/2/3, 4/2/0; crude p = 0.202; PS matched OPPPD group, 7/173 [4.0%], KDIGO stage 1/2/3, 6/0/1; PS matched LPPPD group, 6/173 [3.5%], KIDGO stage 1/2/3, 4/2/0; SMR p = 0.619; PS matching p = 1.000) and postoperative ICU admission (PS matching p = 0.999; SMR p = 0.982) between the two groups (Table 4). The percentage of patients in both groups at each AKI stage is shown in Fig 2. Hospital stay was significantly shorter in the LPPPD group than in the OPPPD group after PS-matching analysis and SMR weighted analysis (16.7 [10.0] vs. 18.7 [9.6] days, p = 0.004; 16.6 [9.9] vs. 18.1 [8.8] days, p = 0.001, respectively) (Table 5).

**Table 4:** Clinical outcomes adjusted by laparoscopic pylorus-preserving pancreaticoduodenectomy. Values are expressed as mean (SD) or n (proportion).

		Crude				SMR weighted set^†^			PS-matched set^†^			
		Event/N (%)	OR	95%CI	P value	OR	95%CI	p value	Event/n (%)	OR	95%CI	P value
**AKI**	OPPPD	37/632 (5.9)	1			1			7/173 (4.0)	1		
	LPPPD	6/177 (3.4)	0.564	0.234–1.359	0.202	1.340	0.423–4.250	0.619	6/173 (3.5)	1.000	0.316–3.163	1.000
**ICU stay**	OPPPD	21/632 (3.3)	1			1			8/173 (4.6)	1		
	LPPPD	7/177 (4.0)	1.198	0.501–2.866	0.685	1.012	0.365–2.808	0.982	7/173 (4.0)	1.000	0.341–2.938	0.999

^†^adjusted for synthetic colloid use, lowest mean arterial pressure, and transfusion

AKI, acute kidney injury; ICU, intensive care unit; SMR, standardized mortality ratio; PS, propensity score; OR, odds ratio; CI, confidence interval; OPPPD, open pylorus-preserving pancreaticoduodenectomy; LPPPD, laparoscopic pylorus-preserving pancreaticoduodenectomy.

**Table 5:** Comparison of hospital stays between two groups. Values are expressed as mean (SD).

	OPPPD	LPPPD	p value
Crude	19.4 (9.7)	16.6 (9.9)	<0.001
PS-matched set	18.7 (9.6)	16.7 (10.0)	0.004
SMR weighted^†^	18.1 (8.8)	16.6 (9.9)	0.001

^†^Weighted t-test after log-transformation of Hospital Stay

OPPPD, open pylorus-preserving pancreaticoduodenectomy; LPPPD, laparoscopic pylorus-preserving pancreaticoduodenectomy; PS, propensity score; SMR, standardized mortality ratio.

## Discussion

In the present study, we demonstrated that there was no significant difference in the incidence of postoperative AKI between the OPPPD group and the LPPPD group. In addition, we showed that no difference was found in the incidence of AKI after PS matching and SMR weighted analysis. However, patients who underwent LPPPD had a shorter period of hospital stay than those who received OPPPD. In multivariable analysis, hypertension, preoperative albumin level, and intraoperative synthetic colloid infusion were associated with the incidence of postoperative AKI.

Studies have shown conflicting results regarding the effects of laparoscopic surgery on postoperative AKI. It has been reported that increased intra-abdominal pressure may cause renal dysfunction by alterations in renal blood flow and ischemia-reperfusion-related oxidative stress during laparoscopic surgery [17–20]. Glomerular filtration rate, effective renal plasma flow, and urine output were decreased in high-intra-abdominal pressure (12 mmHg) group of patients in a study evaluating laparoscopic cholecystectomy [21]. In patients undergoing laparoscopic adrenalectomy, the urine output significantly decreased during pneumoperitoneum [22]. Conversely, a recent study comparing open and laparoscopic liver resection reported that the incidence of postoperative AKI by KDIGO criteria was significantly lower following the laparoscopic procedure than the open technique [14]. Moreover, in a study that measured preoperative and postoperative urinary N-acetyl-beta-D-glucosaminidase of patients undergoing laparoscopic or conventional procedure, there were no differences between the groups, suggesting that laparoscopic surgery is not associated with renal tubular injury [23]. Our present study is in accordance with the aforementioned reports, in which laparoscopic surgery has no significant effect on renal function or occurrence of AKI. Although there are temporary decreases in renal function, this phenomenon does not seem to be clinically significant because urinary output and renal function is restored to normal after the intra-abdominal pressure is reduced to the baseline level, and there was no evidence of microscopic damage to the renal tubule [17, 24].

Less bleeding and less requirement for RBC transfusion may be the possible causes of the similar incidence of postoperative AKI between the two groups in this study, despite prolonged pneumoperitoneum. Hemodilution and transfusion have been reported to be associated with renal dysfunction and AKI [15, 25, 26]. However, although there were statistically significant differences in the amount of infused packed RBCs between the two groups in our study, the difference was not clinically significant, and the transfusion was not an independent risk factor associated with the occurrence of AKI. This is probably due to the fact that significant bleeding did not occur during most of the procedures in both groups.

Rather than the type of surgery, variables including history of hypertension, preoperative albumin level, and synthetic colloid infusion were associated with the occurrence of postoperative AKI. Hypertension is a known risk factor of AKI in patients undergoing general surgery [27, 28]. In a meta-analysis of the association of risk factors including hypertension with AKI, patients with hypertension had a higher risk of AKI at the estimated glomerular filtration rate 60 ml.min^-1^.1.73 m^-2^ or above [27]. This result is consistent with our current study, in which hypertension is significantly associated with the occurrence of AKI.

Lower serum albumin levels are also reported to be associated with perioperative complications including AKI [29–31]. In a study of 756 cardiac transplantation recipients, the serum albumin level was an independent predictor of postoperative AKI (OR 0.34, 95%CI 0.21–0.54) [29]. Another retrospective study of 1309 patients undergoing total knee arthroplasty, an early postoperative (postoperative day 2) albumin level of < 3.0 g.dl^-1^ was revealed to be independently associated with the occurrence of AKI and longer hospital stay [32]. The results of our study show that low preoperative albumin levels were also a risk factor for AKI in PPPD, following either laparoscopic or open surgery.

In our study, Voluven^®^ was used for the administration of synthetic colloids. The main component of Voluven^®^ is hydroxyethyl starch (HES), which is reported to be associated with AKI. In a prospective randomized controlled trial performed by Myburgh et al. [33] which enrolled 7000 patients in the ICU of 32 hospitals, the use of HES was associated with increased risk of kidney injury and renal replace therapy. However, the relationship between HES administration and the incidence of postoperative AKI has not been clarified [34–36]. There was no difference in the occurrence of AKI and need for renal replace therapy between the patients who were administered HES and crystalloids in a meta-analysis which included 13 trials (n = 741), thus the authors concluded that there are not enough data to identify the outcomes related to HES [37]. Although there is still debate, our results add further weight to the claim that HES administration is associated with the development of AKI.

Patients who received LPPPD had shorter hospital stays because of the smaller incision, which allowed the patient to recover quickly and enabled daily life with less pain medication [4]. This can be an advantage of LPPPD in terms of the patient's quality of life and costs.

There are some limitations to our study. As a retrospective study, unexpected biases cannot be excluded. The two groups have differences in their characteristics such as sex, age, underlying diseases and diagnosis. Additionally, patients in the OPPPD group were administered more fluids and had greater transfusions of packed RBCs, which means that a patient in the OPPPD group had a greater likelihood of experiencing volume overload. These could act as confounding factors and affect outcomes of our analysis. Therefore, we attempted to minimize the effects of the confounding factors using PS analysis. The diagnosis for the surgery was not considered as a dependent variable in PS matching and multivariable analysis. Features of the disease including tumour size, lymph node invasion, and differentiation also were not considered in our present study, which may have possibly caused a bias in this study. Moreover, malignancy can be a risk factor for AKI because of various reasons, including prerenal condition, sepsis, nephrotoxins, cytokines, and paraneoplastic conditions [38, 39]. Also, cancer itself or its treatment can cause electrolyte imbalances and tumor lysis syndrome, which are associated with kidney injury and poor prognosis [40–42]. However, although these variables including diagnosis and features of the disease may potentially affect long-term survival, we assumed that these variables had less clinical impact on the occurrence of postoperative AKI according to the type of surgery. In a matched, case-control analysis performed by Song et al. [4] where the difference in oncologic outcome between the OPPPD and LPPPD groups was not significant, there were also no significant differences in overall survival. Because our study was limited to a single center, it has the limitation of tending to target a relatively homogenous or similar population. Further research is warranted to demonstrate the outcomes in heterogenous populations from multiple centers.

We demonstrated that there were no significant differences in postoperative AKI incidence between LPPPD and OPPPD groups in this study. In addition, the LPPPD is not a factor associated with AKI. Therefore, compared with OPPPD, LPPPD may be a promising approach with comparable postoperative outcomes in addition to the advantage of shorter hospital stay.

## Supporting information

S1 File The raw data of the entire study groups. (CSV)
